# The impact of differences in viral entry and trafficking on the mechanism of HIV-1 neutralization

**DOI:** 10.1186/1742-4690-9-S2-P58

**Published:** 2012-09-13

**Authors:** CD Macedo Cincotta, LM Wieczorek, B Barrows, B Brown, L Asher, N Michael, M Marovich, VR Polonis

**Affiliations:** 1 Henry M Jackson Foundation/ U.S. Military HIV Research Program (MHRP), Silver Spring, MD, USA; 2 Termofisher, USA; 3 Military HIV Research Program (MHRP)/ WRAIR, USA

## Background

Cellular entry of HIV-1 occurs through fusion at the plasma membrane (PM) or via endocytosis. PM fusion, resulting in deposit of nucleocapsid into the cytoplasm, typically establishes productive infection. In contrast, entry through endocytosis results in either lysosomal degredation, or productive infection through endosomal membrane fusion. While viral entry and permissivity are known to differ between host cells, the influence of these differences on antibody-mediate neutralization is unknown. Here we explore the relationships between HIV-1 entry, neutralization sensitivity, and the mechanisms by which antibodies act.

## Methods

Differences in viral entry pathways between peripheral blood mononuclear cells (PBMC) and TZM-bl cells were visualized by electron microscopy (EM). Cells were incubated with HIV, with or without antibody, for 1 hour at 4oC, then viral entry was allowed for 5 min at 37oC. Cells were treated with pronase and either separated into cytosolic and membrane fractions, or cultured to assess viral growth and neutralization. The fraction where nucleocapsid localized was determined by antigen capture and microscopy techniques were used to visualize HIV-1 within cells.

## Results

TZMbl cells are 30-fold more efficient at internalizing HIV-1 than PBMC with differences in entry observed between viruses. In TZMbl cells, a greater fraction of p24 localized in the membrane indicating endocytic entry, whereas in PBMC, SF162 localized predominantly in cytosol, indicating PM fusion, while Bal localized equivalently in membrane and cytosolic fractions. Both viruses are neutralized by 2G12 and b12, however, neutralization by 2G12 was not associated with entry inhibition; in fact, after the 5 minutes infection, entry appeared to be enhanced. In contrast, b12 decreased viral entry.

## Conclusion

Our data suggest that, for certain antibodies, inhibition of HIV-1 may occur post-entry, and differences in entry mechanisms may impact neutralization. Experiments to elucidate the role of host restriction and post-entry neutralization on inhibition of HIV-1 are ongoing.

